# Supplementary material for: Pyruvate Dehydrogenase Kinase 1 inhibition mediated oxidative phosphorylation enhancement in cartilage promotes osteoarthritis progression
Source: BMC Musculoskelet Disord. 2023 Jul 20;24:597. doi: 10.1186/s12891-023-06585-6 (PMC10357736; doi:10.1186/s12891-023-06585-6)
Supplement: Supplementary file 1 — Additional file 1. [file 12891_2023_6585_MOESM1_ESM.zip › KEGG.Gsea.1653622432464/KEGG_ONE_CARBON_POOL_BY_FOLATE.html]

Details for gene set KEGG\_ONE\_CARBON\_POOL\_BY\_FOLATE[GSEA]

|  || Dataset | 55457\_collapsed\_to\_symbols.cls.cls #OA\_versus\_normal.cls.cls #OA\_versus\_normal\_repos |
| Phenotype | cls.cls#OA\_versus\_normal\_repos |
| Upregulated in class | normal |
| GeneSet | KEGG\_ONE\_CARBON\_POOL\_BY\_FOLATE |
| Enrichment Score (ES) | -0.22427654 |
| Normalized Enrichment Score (NES) | -0.6275909 |
| Nominal p-value | 0.9259259 |
| FDR q-value | 1.0 |
| FWER p-Value | 1.0 |
Table: GSEA Results Summary

  

Fig 1: Enrichment plot: KEGG\_ONE\_CARBON\_POOL\_BY\_FOLATE      
 Profile of the Running ES Score & Positions of GeneSet Members on the Rank Ordered List

  

| SYMBOL | TITLE | RANK IN GENE LIST | RANK METRIC SCORE | RUNNING ES | CORE ENRICHMENT || 1 | ATIC | 5-aminoimidazole-4-carboxamide ribonucleotide formyltransferase/IMP cyclohydrolase [Source:HGNC Symbol;Acc:HGNC:794] | 401 | 0.706 | 0.1272 | No |
| 2 | SHMT2 | serine hydroxymethyltransferase 2 [Source:HGNC Symbol;Acc:HGNC:10852] | 1518 | 0.426 | 0.1362 | No |
| 3 | MTHFS | methenyltetrahydrofolate synthetase [Source:HGNC Symbol;Acc:HGNC:7437] | 2155 | 0.343 | 0.1638 | No |
| 4 | FTCD | formimidoyltransferase cyclodeaminase [Source:HGNC Symbol;Acc:HGNC:3974] | 3064 | 0.244 | 0.1480 | No |
| 5 | TYMS | thymidylate synthetase [Source:HGNC Symbol;Acc:HGNC:12441] | 4328 | 0.126 | 0.0782 | No |
| 6 | MTHFR | methylenetetrahydrofolate reductase [Source:HGNC Symbol;Acc:HGNC:7436] | 4334 | 0.125 | 0.1059 | No |
| 7 | DHFR | dihydrofolate reductase [Source:HGNC Symbol;Acc:HGNC:2861] | 5805 | 0.008 | -0.0064 | No |
| 8 | MTHFD2L | methylenetetrahydrofolate dehydrogenase (NADP+ dependent) 2 like [Source:HGNC Symbol;Acc:HGNC:31865] | 7789 | -0.141 | -0.1287 | No |
| 9 | AMT | aminomethyltransferase [Source:HGNC Symbol;Acc:HGNC:473] | 9021 | -0.234 | -0.1719 | Yes |
| 10 | MTR | 5-methyltetrahydrofolate-homocysteine methyltransferase [Source:HGNC Symbol;Acc:HGNC:7468] | 9213 | -0.249 | -0.1309 | Yes |
| 11 | ALDH1L1 | aldehyde dehydrogenase 1 family member L1 [Source:HGNC Symbol;Acc:HGNC:3978] | 9302 | -0.256 | -0.0804 | Yes |
| 12 | SHMT1 | serine hydroxymethyltransferase 1 [Source:HGNC Symbol;Acc:HGNC:10850] | 9727 | -0.294 | -0.0474 | Yes |
| 13 | GART | "phosphoribosylglycinamide formyltransferase, phosphoribosylglycinamide synthetase, phosphoribosylaminoimidazole synthetase [Source:HGNC Symbol;Acc:HGNC:4163]" | 9861 | -0.307 | 0.0112 | Yes |
| 14 | MTHFD1 | "methylenetetrahydrofolate dehydrogenase, cyclohydrolase and formyltetrahydrofolate synthetase 1 [Source:HGNC Symbol;Acc:HGNC:7432]" | 10249 | -0.346 | 0.0587 | Yes |
| 15 | MTHFD2 | "methylenetetrahydrofolate dehydrogenase (NADP+ dependent) 2, methenyltetrahydrofolate cyclohydrolase [Source:HGNC Symbol;Acc:HGNC:7434]" | 12239 | -0.656 | 0.0513 | Yes |
Table: GSEA details [plain text format]

  

Fig 2: KEGG\_ONE\_CARBON\_POOL\_BY\_FOLATE      
 Blue-Pink O' Gram in the Space of the Analyzed GeneSet

  

Fig 3: KEGG\_ONE\_CARBON\_POOL\_BY\_FOLATE: Random ES distribution      
 Gene set null distribution of ES for **KEGG\_ONE\_CARBON\_POOL\_BY\_FOLATE**
